# The Differential Outcomes Effect in Pigeons (*Columba livia*): Is It Truly Anticipatory?

**DOI:** 10.1371/journal.pone.0150510

**Published:** 2016-03-02

**Authors:** Marijn Kouwenhoven, Michael Colombo

**Affiliations:** Department of Psychology, University of Otago, Dunedin, New Zealand; Universidad de Chile, CHILE

## Abstract

We used delay-interval interference to investigate the nature of the differential outcomes effect (DOE) in pigeons. Birds were trained on a delayed matching-to-sample (DMS) task under either common outcome or differential outcome conditions, and then presented with visual interference during the delay period. Consistent with previous literature, the common outcomes birds were slower to learn the DMS task than the differential outcomes birds. The common outcome birds were also more impaired by the visual interference than the differential outcomes birds. Our findings are consistent with the view that the birds trained with common outcomes were likely remembering the sample stimulus during the delay period, and hence were disrupted by the visual interference, whereas the birds trained with differential outcomes were likely relying on the different emotional reactions elicited by the different outcomes to guide their choice behaviour, and hence were less affected by the visual interference. Our findings suggest that the DOE is not truly evidence of anticipatory mediation of short-term retention in pigeons, but rather emotionally driven decision making, which is not truly anticipatory in nature.

## Introduction

According to Zentall and Sherburne [1], the differential outcomes effect (DOE) is considered to be one of the most integral foundations of animal cognition research. The DOE refers to a phenomenon where subjects learn tasks faster when different rewards are uniquely associated with different responses [2, 3]. Not only are tasks learned faster, but rewarding responses differentially also substantially decreases the rate of forgetting over a long period of time [4]. So powerful is the DOE that even if the task is designed so that one of the outcomes is not necessarily rewarding, for example sample A predicts food but sample B predicts a light or even the absence of food, learning will still be superior to situations where the same reward is provided for the different responses [4]. Trapold [2] suggested that when trained with differential outcomes, the subject learns to anticipate a specific reward. In other words, animals form distinct expectancies based on the different sample-reinforcer associations. These expectancies act as additional cues for the subject to base its decision when it has to make a choice, and these additional cues lead to faster learning and better retention during a delay [2, 3].

Grant and Kelly [5] argue that whilst research using DO paradigms provides strong evidence for anticipatory short-term memory mediation, there are still questions about the nature of the expectancies that differential outcomes evoke, and whether these expectancies are truly anticipatory. One possibility is that the expectancies are visual representations of the different outcomes, for example an illuminated food hopper with food, and an illuminated food hopper with no food available. A second possibility, however, is that the different outcomes come to elicit drastically different emotional reactions in a subject. For example, if sample A is associated with an illuminated food hopper with food, and sample B is associated with an illuminated food hopper with no food, one would expect sample A and sample B to evoke different emotional reactions. Specifically, sample A should evoke a positive emotional reaction, and sample B should evoke a negative emotional reaction. Grant and Kelly [5] suggest that it is these emotional reactions and not a visual representation of the outcome that persists during a delay and guides choice behaviour, and so they do not constitute as a true instance of anticipatory memory.

In the current study, we applied the principle of modality-specific interference [6] and used visual interference to determine whether the expectancies that a DO paradigm evokes are visual or emotional in nature. One group of birds was trained under a common outcomes (CO) condition in which the same reward followed a correct response to either of two possible to-be-remembered stimuli, and another group of birds was trained on a differential outcomes (DO) condition in which a correct response to one of the to-be-remembered stimuli was followed by a reward, and a correct response to the other was followed by the absence of a reward. In the CO condition there is no possibility of using the expectancy of reward to assist in retention, and so the birds must be remembering the sample stimulus during the delay period.

The situation for the DO condition, however, is somewhat different to that of the CO condition, In the DO condition the birds are able to rely on outcome expectancy by either storing visual representations of the expected outcomes, or by relying on the emotional reaction to the sample stimulus. Based on the principle of modality-specific interference that visual interference affects the retention of visual information [7], if visual interference impairs performance on both the CO and DO tasks to the same degree then we would consider this as support for the idea that outcome expectancies take the form of visual representations. On the other hand, if visual interference impairs performance on the CO task more than on the DO task, we would consider this as support for the idea that outcome expectancies elicit different emotional reactions that guide choice behaviour.

## Materials and Methods

### Subjects

The subjects were 20 experimentally naïve pigeons (*Columba livia*) randomly assigned to either the common outcomes (CO) or differential outcomes (DO) group. The pigeons were housed in individual cages in a colony room maintained at 20°C, with 12 hours off, 12 hours on lighting conditions. They were maintained at 80–85% of their free-feeding weight and trained five to six days a week. Upon conclusion of the current study, all pigeons were used as subjects in other experiments. This research was approved by the University of Otago Animal Ethics Committee (approval number 61–13).

### Apparatus and Stimuli

The pigeons were trained in four standard operant chambers. Each chamber was constructed of black interior walls with a front Perspex panel providing access to a 15-inch LCD touchscreen monitor. The panel had six 60mm by 60mm square holes arranged in a 2-row by 3-column grid. The stimuli were displayed on the monitor and appeared only on the top row of holes. The monitor was connected to a PC computer that was responsible for presenting the stimuli and recording responses. Wheat reward was delivered by an illuminated (when active) food hopper located 210mm below the middle-top hole in the Perspex panel. The stimuli used were two black and white photos, one of a flower and one of a skateboarder (hereafter referred to as “skater”).

### Procedure

The delayed matching-to-sample (DMS) procedure was as follows. A sample stimulus (either “flower” or “skater”) appeared in the centre hole. Three pecks to the sample turned it off and initiated a delay period. At the end of the delay both the “flower” and “skater” comparison stimuli were presented on the side holes. A response to the stimulus that had appeared as the sample was considered correct. A response to the nonmatching comparison stimulus was considered an incorrect response.

For the CO birds, a correct response always resulted in 2 sec of access to wheat via an illuminated hopper. For the DO birds, a correct response following the “skater” sample resulted in 2 sec access to wheat via the illuminated hopper, whereas a correct response following the “flower” sample illuminated the hopper light, but no food was presented. All correct responses were followed by a 10-sec inter-trial interval (ITI). Incorrect responses initiated a 5-sec time-out accompanied by a low tone (2Hz), followed by the 10-sec ITI. A correction procedure was in place at all times and incorrect responses were followed by a repetition of the same trial until the animal made the correct response. A session consisted of 64 trials. Each sample stimulus was presented 32 times in a quasi-randomized order. Which stimulus appeared as the sample stimulus, as well as the left-right positions of the comparison stimuli, were fully randomized within a session.

The pigeons were initially trained on the DMS task with a 0-sec delay period until they reached a criterion of two consecutive sessions with 52/64 (81.25%) correct responses. The delay was then increased to 1 sec and the birds trained until they again attained a criterion of two consecutive sessions with 52/64 (81.25%) correct responses. In the final phase of acquisition the delay was increased to 3 sec and the birds trained until they attained a criterion of 50/64 (78.1%) correct responses for two consecutive sessions. To ensure that the pigeons were learning both sample-comparison associations to a satisfactory level there was an additional criterion at each delay phase consisting of performance of at least 25/32 (78.1%) correct in a session for each sample stimuli. Once the pigeons reached criterion on the 3-sec delay they were moved on to the main interference phase of the experiment.

For interference testing the delay between sample presentation and comparison presentation was increased to 5 sec. During the entire delay period we introduced visual interference by illuminating the chamber with a houselight as well as changing the monitor display from a black screen to a white screen. Visual interference occurred on a random half of the trials within a session, spread equally across trials where “skater” or “flower” was the sample. Interference testing ran for a total of 14 sessions.

## Results and Discussion

Of the 10 DO birds, three eventually refused to respond to the sample that predicted no reward, and hence failed to complete the DMS task with a 0-sec delay and were removed from the study. Of the 10 CO birds, five required more than 80 sessions to complete acquisition, and due to time constraints were unable to test the birds under the interference condition.

Consistent with previous literature, the DO birds learned the DMS task faster than the CO birds. A two-way repeated-measures ANOVA with Condition (DO vs CO) as the between-subjects factor and Delay (0 vs .5 vs 1 vs 3) as the within-subjects factor revealed a significant effect of Condition, *F*(3, 42) = 13.51, *p* < .001, a significant effect of Delay, *F*(3, 42) = 13.64, *p* < .001, and a significant Condition x Delay interaction effect, *F*(3, 42) = 13.51, *p* < .001. On average, the DO birds were faster to learn the DMS task than the CO birds at the 0-sec (5.14 sessions vs 14.50 sessions), .5-sec (2.70 sessions vs 7.50 sessions), 1-sec (2.71 sessions vs 11.80 sessions), and 3-sec (3.00 sessions vs 29.56 sessions) delays.

**Fig 1:**
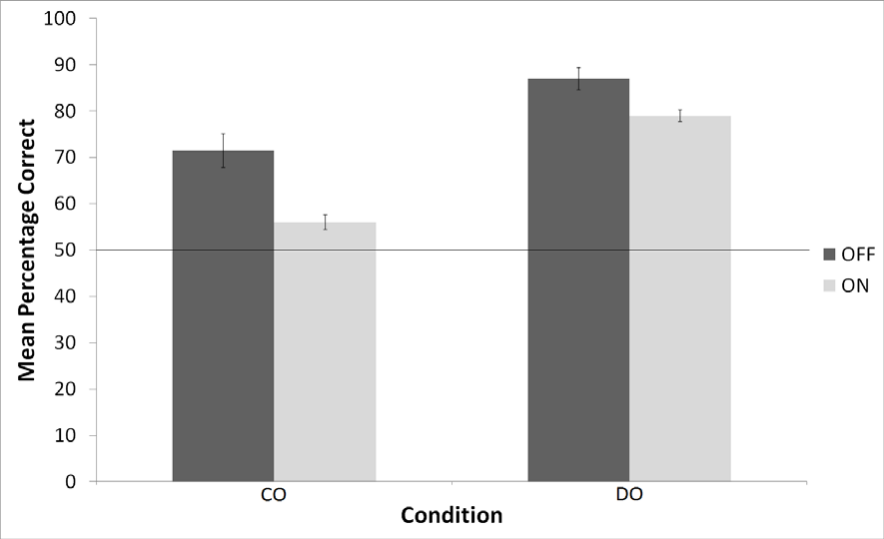
Mean percentage correct for Interference OFF and Interference ON trials for the CO and DO birds. The horizontal line indicates chance levels of performance.

Performance on the interference test is shown in Fig 1. The data were subjected to a repeated-measures ANOVA with Condition (CO vs DO) as the between-subjects factor and Interference (OFF vs ON) as the within-subjects factor. We found a significant Condition x Interference interaction, *F*(1, 10) = 5.20, *p* < .05, which is due to the fact that the interference impaired performance of the CO birds more than the DO birds. Whilst the CO birds were more impaired by the interference than the DO birds, we also found a significant main effect of Interference, *F*(1, 10) = 51.79, *p* < .001, which suggests that the DO birds were still affected by the visual interference.

**Fig 2:**
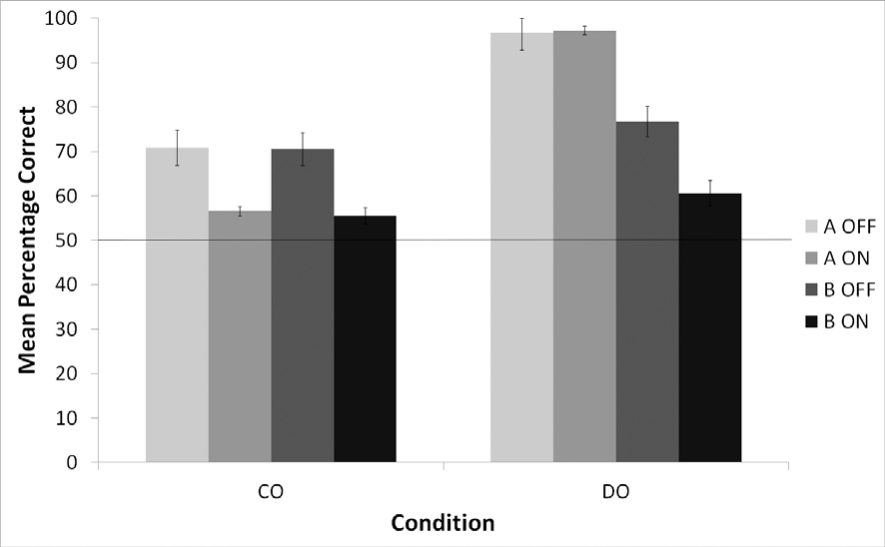
Mean percentage correct for the “skater” and “flower” sample stimuli during Interference OFF and Interference ON trials. The horizontal line indicates chance levels of performance.

To further investigate the effect of interference, we separated the data according to their corresponding trial types. The results are shown in Fig 2. Performance on trials where the sample stimulus was “skater” are shown separately from performance on trials where the sample was “flower”. Recall that for DO birds “skater” was rewarded and “flower” was not, whereas for CO birds, both “skater” and “flower” were rewarded. The data for the CO birds was analyzed using a two-way repeated-measures ANOVA with Interference (OFF vs ON) and Stimulus (“skater” vs “flower”) as factors. There was a significant effect of Interference, *F*(1, 4) = 41.06, *p* < .05, but no effect of Stimulus, *F*(1, 4) = .15, *p* = .72, and no Interference x Stimulus interaction effect, *F*(1, 4) = .01, *p* = .94, suggesting that the effect of Interference was the same for both the “skater” and “flower” stimuli. The DO birds’ data was also subjected to a two-way repeated-measures ANOVA with Interference (OFF vs ON) and Stimulus (“skater” vs “flower”) as factors. There was a significant effect of Interference, *F* (1, 6) = 10.36, *p* < .05, and in contrast to the outcome for the CO birds there was also a significant effect of Stimulus, *F*(1, 6) = 185.84, *p* < .001, and a significant Interference x Stimulus interaction effect, *F*(1, 6) = 8.22, *p* < .05. The significant interaction effect is the result of the fact that the visual interference had no effect when the sample was “skater”, but severely impaired the DO birds’ performance when the sample was “flower”.

In summary, we found that the CO birds were more impaired by the visual interference than the DO birds. The DO birds were also affected by the visual interference, but only when the non-rewarded “flower” was the to-be-remembered stimulus–the visual interference had no effect when the rewarded “skater” was the to-be-remembered stimulus. Remarkably, the performance of the DO birds on trials where “flower” was the stimuli was still superior to the performance of the CO birds on both “skater” and “flower” trials. Given that the DO birds were not rewarded for responding correctly on the “flower” trials whereas the CO birds were rewarded after all correct choices, the superior performance of the DO birds is noteworthy. We consider the overall superior performance of the DO birds as another piece of evidence that rewarding responses differentially results in improved performance on a DMS task.

As for our hypotheses, our results are consistent with the notion that outcome expectancies elicit different emotional reactions that guide choice behaviour. If the DO birds were storing visual representations of the expected outcomes then visual interference should have impaired the DO birds’ performance with both the “skater” and “flower” stimuli, and clearly that was not the case. The fact that the visual interference had no effect on the “skater” (rewarded) stimulus is fully consistent with the view that under the DO condition the animals are not anticipating the stimulus, but rather bridging the delay period by remembering the emotional reaction associated with the stimulus.

One possibility that should be considered is that the content of the short term memory could be a combination of the visual image of the sample and the emotional reaction elicited by it. However, the main idea behind the DOE is that the differential outcomes paradigm allows animals to form distinct expectancies based on the different sample-reinforcer associations. These expectations act as *additional* cues for the subject to base its decision when it has to make a choice. If both the CO and DO birds’ short term memory for the sample is visual in nature, and it seems reasonable that it is, then it seems logical that any additional cue would be most salient if it was not visual in nature. As such we believe our data support the view that the nature of the additional cue might be an emotional reaction rather than a visual image.

We do recognize that the fact that the visual interference did have an effect on the “flower” (non-rewarded) stimulus initially appears to be at odds with the idea of evoked emotional reactions. However, Browning, Overmier, and Colombo [8] suggested that pigeons can develop an aversion to responding towards a stimulus that is associated with a negative event (in this case, not getting food). Furthermore, the correction procedure that we used in our experiment ensured that, in addition to “flower” signalling that there was no opportunity for reward, repetitions of “flower” trials also prolonged the time to a potential “skater” trial and subsequent reward. The aversion that the DO birds might have felt towards “flower” could have made them reluctant to respond at all, and after not responding for a while, the DO birds would default to remembering “skater” during the delay period and then incorrectly choosing “skater” during the comparison period. This behaviour would obviously lower the percent accuracy on “flower” trials, as well as make “flower” trials susceptible to visual interference. If this is indeed the case then our data support the idea that differential outcomes elicit different emotional reactions, as these reactions were clearly guiding the DO birds’ behaviour.

A potential confound that should be addressed is that the level of accuracy on the non-interference trials was not the same for the CO and the DO birds. As can be seen in Fig 1, the DO birds’ performance on Interference OFF trials was significantly superior to the performance of the CO birds. A lower baseline performance on Interference OFF trials could also account for the bigger disruption experienced by the CO birds on the Interference ON trials. One solution to this problem would be to equate the levels of performance in the CO and DO conditions–that is, train the CO birds until their performance is on par with that of the DO birds. Such a solution is difficult to achieve, however, because the DO birds are likely to always out-perform the CO birds–such is the very nature of the DOE. That said, there was one CO bird whose performance matched that of one DO bird in the interference OFF condition (81.25% and 80.13%, respectively). Despite being matched in the interference OFF condition, in the interference ON condition the performance of the CO bird was 60.04% whereas that of the DO bird was 82.3%. Thus, in the one case where the performance between a CO and DO bird was matched in the interference OFF condition, the DO bird still showed, consistent with our hypothesis, superior performance in the interference ON condition compared to the CO bird.

Another potential confound is that we failed to counterbalance the roles of the two sample stimuli in the DO condition. Correct responses to the “flower” sample were always unrewarded, and correct responses to the “skater” sample were always rewarded. We do not believe that this was an issue, however, as there was no difference in performance on the two samples in the CO condition.

## Conclusion

Our findings are consistent with the view that the CO birds were likely remembering the sample stimulus during the delay period, and hence were disrupted by the visual interference. In contrast, the DO birds were likely being guided by the different emotional reactions elicited by the different outcomes, and hence were less affected by the visual interference. Our findings suggest that the DOE is not truly evidence of anticipatory mediation of short-term retention in pigeons, but rather that subjects’ behaviour is simply guided by emotional reactions elicited by the different samples and outcomes, as proposed by Grant and Kelly [5].
